# Self-powered intelligent pulse sensor based on triboelectric nanogenerators with AI assistance

**DOI:** 10.3389/fbioe.2023.1236292

**Published:** 2023-09-15

**Authors:** Yifei Tian, Cong Hu, Deguang Peng, Zhiyuan Zhu

**Affiliations:** ^1^ Chongqing Key Laboratory of Nonlinear Circuits and Intelligent Information Processing, College of Electronic and Information Engineering, Southwest University, Chongqing, China; ^2^ Guangxi Key Laboratory of Automatic Detecting Technology and Instruments, Guilin University of Electronic Technology, Guilin, China; ^3^ Chongqing Megalight Technology Co., Ltd., Chongqing, China

**Keywords:** artificial intelligence, triboelectric nanogenerators, pulse detection, TENG, AI

## Introduction

Pulse is a commonly observed physiological phenomenon that provides crucial information of the cardiovascular system and other physiological processes (Suguna and Veerabhadrappa, 2019). Pulse measurement provides a physiological reference for blood pressure, blood flow, and other physiological tests. Additionally, the pulse wave itself serves as a valuable indicator of diagnostic information (Khandelwal et al., 2019). Indian Ayurvedic and Chinese traditional medicines depend on wrist pulse wave signal parameters for diagnosis of human health and potential diseases. Pulse detection is a simple, quick, and easy-to-use physiological health indicator, which is critic in many medical applications (Cheng et al., 2019). However, traditional pulse detection methods are predominantly reliant on subjective perception and touch by healthcare providers, leading to inaccuracies in diagnostic outcomes. Pulse and other human characteristics are subject to change over time and with varying environmental factors. As a result, individual measurements might result in additional measurement errors (Wang et al., 2015). Modern pulse meters and pulse oximeters still have limitations. As an example, pulse measuring devices necessitate a consistent electric power source or battery (Liu et al., 2019), imposing substantial limitations on their applicability in specific environments or use cases. This ultimately hampers their mobility and accessibility. Furthermore, the high cost of high-end pulse measuring devices is not economically viable for specific medical institutions or patient populations (Liu et al., 2019).

**FIGURE 1:**
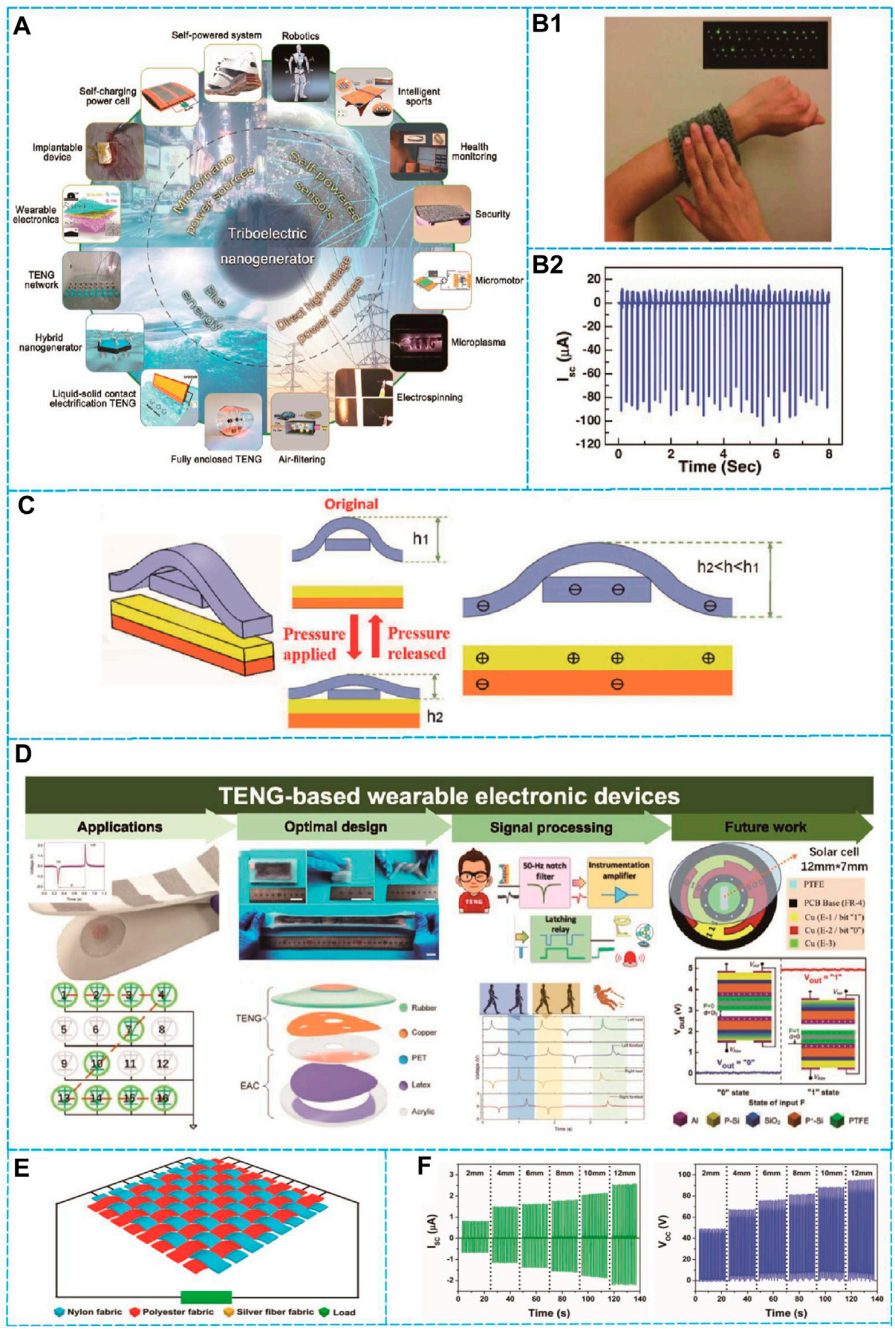
Application of pulse detection device based on AI-TENG in previous reports. **(A)** The utilization of Triboelectric Nanogenerator (TENG)-based self-powered sensors finds applications in diverse domains such as biomedicine, the Internet of Things (IoT), and blue energy systems. This information has been adapted with authorization from the reference cited as (Liu et al., 2020b). **(B1)** Photographic evidence illustrates the capability of the Self-Powered Stretchable Electronic Skin for Health Monitoring (SEHT) to energize Light-Emitting Diodes (LEDs) through the process of energy harvesting from tactile interactions with the wrist-worn SEHT device. **(B2)**/sc from tapping on the wrist-worn SEHT. **(B1,B2)** reproduced with permission from ref (Lv et al., 2017). **(C)** Schematic representation depicting the cross-sectional perspective of an individual unit within the Woven Pressure Sensor (WCSPS). **(C)** reproduced with permission from ref (Zhang et al., 2013). **(D)** Rich functions of TENG-based health detection equipment. **(D)** reproduced with permission from ref (Wang, 2017). **(E)** Structure and fabrication of the woven-structured triboelectric nanogenerator (W-TENG). **(F)** Structure and electrical outputs of the W-TENG under nondeformation mode. **(E,F)** reproduced with permission from ref (Zhou et al., 2014).

As technology continues to evolve, more precise and objective pulse detection methods have emerged. In 2012, Wang’s team (Xu et al., 2021) successfully combined the triboelectric effect and the electrostatic induction principle to create a triboelectric nanogenerator (TENG) that collects mechanical energy from the environment (Fan et al., 2012). They affixed PET (Polyethylene terephthalate) and Kapton films, subsequently applying an electroplated metal electrode layer onto the surface to fabricate a TENG. Subsequent research by experts and scholars optimized TENG’s structure, materials, and production process. Further, using flexible printing technology, Meng’s team (Xu et al., 2018a) achieved the first large-scale TENG production. Additionally, Wang’s team (Meng et al., 2013) used plasma technology to increase the TENG’s peak power density to 315 w/m^2^, which improved its overall performance by almost 25 times and laid a foundation for future TENG theoretical research by providing a new method to increase the dielectric layer’s charge density (Li et al., 2023a). Since its invention, TENG based on Maxwell displacement current have shown explosive growth (Liu et al., 2019), from the basic mechanism research (Meng et al., 2013; He et al., 2018) to multi-functional practical applications (Wang et al., 2014; Li et al., 2023a). Due to its exceptional voltage or current responsiveness to vibration, it has emerged as one of the most auspicious contenders for realizing self-powered systems (Wang, 2020). TENG offers several advantages such as being lightweight, low-cost (Liu et al., 2020a), easy to manufacture, versatile, and having high toughness in comparison to biofuel cells, thermoelectric devices, electromagnetic, and piezoelectric generators (Liu et al., 2020b). Moreover, TENG’s friction material can be almost any existing material, making it suitable for a wide range of applications (As depicted in A of Figure 1) and solving most of the problems with current pulse detection devices. In summation, the realm of pulse detection applications holds significant promise for the utilization of TENG due to its immense potential (Yu et al., 2019).

The TENG-based pulse detection sensor is more sensitive compared to traditional or larger analytical instruments. Upon contact with the patient’s skin, the TENG sensor detects the subtle vibrations of the pulse signal (Li et al., 2021a). The interaction of the friction plate and the electrode during the contact and separation process facilitates charge transfer, culminating in the production of an electrical energy signal originating from these minute vibrations. The generated electrical signals can be collected and analyzed to derive characteristic parameters of the pulse signal (Liu et al., 2020c), including heart rate. Furthermore, the TENG-based pulse detection sensor is a self-powered induction device with high-output signal capability (Yang et al., 2015), which facilitates real-time monitoring and allows for the immediate detection and monitoring of a patient’s pulse without appointments or queues (Zou et al., 2019). This attribute holds particular significance in scenarios requiring emergency and critical monitoring (Xu et al., 2021).

Nevertheless, all biological sensors inherently encounter irregular signal noise, presenting a hurdle to precise detection and analysis. Our study employs machine learning optimization algorithms and models to enhance the precision and effectiveness of TENG-based pulse detection sensors (Jiang et al., 2023). TENG sensor signals often necessitate preliminary processing procedures, encompassing tasks such as filtering, denoising, and compensation (Xu et al., 2022). We can leverage machine learning technology to optimize the parameters of these preprocessing algorithms and improve the quality and accuracy of the signals (Xiong and Lee, 2019). TENG sensors output a time-series signal, which necessitates its conversion to a pulse signal via signal processing and classification algorithms (Lv et al., 2017). Machine learning technology can aid in designing more precise and robust classification algorithms, which can enhance signal classification accuracy (Jiang et al., 2023). Moreover, by meticulously scrutinizing extensive pulse data and relevant parameters via machine learning, we can devise a prognostic model adept at discerning pulse characteristics across varied scenarios (Zhang et al., 2013). This, in turn, aids medical professionals in enhancing their diagnostic capabilities and monitoring patients’ wellbeing (Yuan et al., 2021).

Within this paper, we delve into the efficacy of TENG-based pulse measurement sensors for blood pressure monitoring and diagnosis (Zhou et al., 2022), bolstered by the assistance of artificial intelligence (Liu et al., 2023). Our paper aims to report on the use of these sensors, along with artificial intelligence technology, in enhancing blood pressure monitoring and diagnosis (Zhang et al., 2022).

### Self-powered TENG pulse detection

The technology of Triboelectric Nanogenerators (TENG) has promising potentials in pulse detection. By harnessing the mechanical motion of human pulse, TENG generates electricity (Chen et al., 2020). Its unique wearability and portability, coupled with its independence from external power sources, render it an optimal selection for real-time pulse detection and monitoring (Zhang et al., 2014). Han et al. introduced a flexible self-powered ultra-sensitive pulse sensor (SUPS) that employs frictional-electric active sensor technology (Ouyang et al., 2017), demonstrating superior output performance (1.52 V), high peak signal-to-noise ratio (45 dB), long-term endurance (107 cycles), and affordability. The TENG placed on the wrist detects changes in wrist pulse pressure that correspond to human health, which it senses through the electrical signals produced (Lai et al., 2017). Ying-Chih Lai et al. developed an SE-TENG that has a simple structure and enhanced flexibility, making it highly convenient for harvesting energy from human skin. The device comprises a basic groove structure with PDMS film and ITO-coated PET film merged together. The efficiency of the apparatus relies on the magnitude and profundity of the groove structure. Increasing the depth of the grooves would cause the output signal of the pulse sensor to decrease linearly (Meng et al., 2019). Conversely, reducing the groove’s depth would increase the contact area between the PDMS film and the ITO electrode, resulting in substantial charge variation (Li et al., 2023b). When the groove structure feels pulse pressure, the bottom PDMS layer interacts more with the ITO electrode, transmitting charge from the ground electrode. Nonetheless, in the event of an electrostatic equilibrium between the frictional-electric films, charge transfer remains dormant in the absence of pulse pressure. The wrist pulse acts constantly on the PDMS film, creating a back-and-forth current flow. The schematic diagram and output pulse wave of the single-electrode pulse sensor are depicted in B1-B2 of Figure 1 (Suguna and Veerabhadrappa, 2019). Meng et al. developed a self-powered, flexible, woven pressure sensor (WCSPS) to non-invasively diagnose hypertension-related diseases through pulse measurement (Wu et al., 2019), as depicted in C of Figure 1. The device comprises interlaced polymer nanowires (Seo et al., 2016) in a frictional electric material. The WCSPS offers an exceptional sensitivity, with a response time of <5 ms, and a weaving structure that is 1.64 times greater in effective contact area and electrical output compared to an unwoven structure (Ardila et al., 2019). The WCSPS demonstrated reliable performance, maintaining sensor capabilities after 40,000 repeated motion cycles (Su et al., 2016). The WCSPS was applied to measure blood pressure from 100 participants representing diverse ages and health statuses ranging from 24 to 82 years old. Differences between the results from the WCSPS and those from cuff-based devices ranged from 0.87% to 3.65%. Liu et al. proposed a straightforward and cost-effective approach for monitoring subtle biological signals, such as respiration and pulse, to achieve a highly sensitive, self-powered, and flexible pressure sensor based on TENG. This was achieved by synergizing the characteristics of PDMS and thermally expandable microspheres (Li et al., 2019). By spin-coating a mixture of thermally expandable microspheres and PDMS on a planar substrate, a large-area patterned friction-charged thin layer was created (Li et al., 2019). Upon heating, the microspheres expanded, forming microstructures on the original flat PDMS surface. As the weight percentage of added thermally expandable microspheres increased, the sensor’s sensitivity also increased, achieving a maximum sensitivity of 1 mV/Pa at a weight percentage of 150%. A theoretical model for analyzing the output voltage was proposed, which exhibited good agreement with experimental results, highlighting substantial potential in pulse monitoring.

This study revealed that utilizing TENG for pulse measurement is an effective approach to health monitoring (Li et al., 2019), which could provide a competing alternative to currently existing complex monitoring systems (As depicted in D of Figure 1). Looking ahead to medical applications, enhancing the electrostatic charge effect of the device and augmenting the dielectric constant of functional materials can be achieved through the chemical incorporation of nanoparticles within an electrostatic composite layer, encompassing nanoparticles, nanotubes, and nanowires (Yao et al., 2022). Material-wise, Combining nylon nanofibers with composite materials such as polyvinylidene fluoride-silver nanowires and polytetrafluoroethylene (PTFE) is a promising method to enhance the electrostatic charge of TENG devices. Additionally, material selection can be optimized based on specific application requirements (Wang, 2017). Nevertheless, notwithstanding the promising attributes of the TENG-based pulse detection device, it confronts specific limitations during the data processing phase. These limitations consist of a large quantity of data, interference from noise, and signal complexity. As a result of these limitations, the extraction of valuable pulse information using traditional data processing methods is arduous (Zhou et al., 2014). It is imperative to surmount these constraints by implementing robust machine learning algorithms and models, that will in turn, enhance the efficiency, and precision of data processing (Yuan et al., 2021). By intelligently analyzing, filtering, and identifying the pulse signal, the performance of the pulse detection device can be considerably improved.

### The prospect of machine learning in TENG-based pulse detection device

The integration of biosensors with machine learning (ML) in medical applications enhances the ability of healthcare systems and decision makers to process information, insights, and environmental data, enabling personalized medicine that minimizes misdiagnosis or late diagnosis (Zhang et al., 2017). For example, Google and Northwestern University have developed an AI model for lung cancer detection in which more than 42,000 CT scan images were used for training (Zhou et al., 2022). The model is able to detect cancer in a single CT scan—with 5% higher accuracy than human experts and exhibits a 9.5% higher detection rate than radiologists in predicting cancer risk 2 years in advance (Riera et al., 2012). Specifically, machine learning has heightened the precision of pulse detection devices, empowering their algorithms to discern subtle pulse signal nuances through the assimilation and analysis of extensive pulse data. This, in turn, culminates in refined detection outcomes. Secondly, machine learning further enhances pulse detection devices’ real-time capability by enabling its algorithm to process and instantly analyze data from TENG sensors to swiftly detect and recognize pulse signals, permitting real-time monitoring and feedback. Lastly, machine learning offers adaptability by allowing pulse detection devices to adaptively analyze and process pulse signals through individual physiological characteristics and environmental modifications, resulting in more personalized and precise results.

Yao et al. proposed the TENG-Cat-System, a human self-propelled catalytic promotion system that improves cancer treatment by studying the electrostatic preorganization effect in natural enzymatic catalysis processes (Ardila et al., 2019; Liu et al., 2023). Enhancing the generation of reactive oxygen species (ROS) using nanocatalysts is essential to improve cancer therapeutic efficacy. The TENG-Cat-system utilizes a one-dimensional porphyrin covalent organic framework (COF) on carbon nanotubes (CNTs) to generate ROS, and markedly enhances its peroxidase-like activity under the human inherent electric field. Employing machine learning for data analysis in conjunction with physiological parameters, the system assists physicians in predicting patient disease progression, therapeutic response, and prognosis. Yuan et al. produced an additive-manufactured, low-cost, and disposable 3D-printed acoustic triboelectric nanogenerator (A-TENG) that collects low-frequency acoustic energy in academic and medical fields (Li et al., 2019; Zhang et al., 2022). Under a 100 dB sound pressure level, the system generates a power output of 4.33 mW. The acoustic resonator system has the capacity to continuously power up to 72 LEDs and a commercial calculator, highlighting its potential as a device power source. Augmented with an artificial intelligence chip that leverages pre-trained neural networks to identify and process the converted electrical signals of A-TENG, the system showcases substantial potential for an intelligent Internet of Things (Zeng et al., 2020).

Currently, the monitoring of high-risk or diseased patients’ health primarily depends on clinical observations and laboratory diagnostic tools that can be expensive and inconvenient (Lin et al., 2020). To overcome these limitations, individual sensing data such as pulse rate, respiratory rate, or blood pressure signals are integrated into an “early risk rating” that aids in health management. Machine learning plays a significant role in this integration process (Zhang et al., 2014). For instance, Riera and colleagues proposed a method that combines EEG and EMG signals for stress detection, which considerably increases the classification accuracy from 79% to 92% through data fusion. This method utilizes periodic reuse and quantitative sensing, providing a more comprehensive understanding of the relationship between medical conditions and physical and mental health, leading to more valuable diagnosis and predictions (Su et al., 2016; Ouyang et al., 2017). Furthermore, Zeng and his team used diverse machine learning techniques to develop their epidermal electronic system (EES) to monitor and predict levels of mental fatigue, achieving an impressive 89% prediction accuracy, which highlights the vast potential of machine learning in the medical field (Lai et al., 2017).

These instances underscore the prowess of machine learning in efficiently processing data and discerning patterns. Possessing the ability to use diverse training models and optimization algorithms, the pulse detection device employing TENG holds great promise as an essential tool in the health monitoring industry (Meng et al., 2019). By analyzing the basic properties of pulse waves and considering clinical requirements and physiological significance, we recommend utilizing the wavelet transform modulus maxima detection algorithm alongside morphology operations to improve the performance of the pulse detection device (Li et al., 2023b). Moreover, optimizing the algorithm through wavelet threshold denoising can enhance the detection and location of extreme points of pulse signals, even in situations with severe noise interference (Wu et al., 2019), baseline drift, or other forms of interference (Seo et al., 2016). Furthermore, in the context of pulse signal analysis, Recurrent Neural Networks (RNNs) process each step of pulse data, learning temporal patterns to predict future features or detect anomalies. RNNs build long dependencies in sequential data, but traditional RNNs suffer from vanishing gradients when handling long sequences. To overcome this, variants like Long Short-Term Memory (LSTM) and Gated Recurrent Units (GRU) have been introduced, using gating mechanisms to effectively capture and retain information in lengthy sequences (Leng et al., 2023). This enhances RNNs’ performance in analyzing pulse signals and other sequential data.

## Discussion

Over the past few years, considerable progress has been made in the medical detection and clinical diagnosis field, utilizing pulse detection devices that rely on the Triboelectric Nanogenerator (TENG) technology. Notwithstanding, several challenges persist that jeopardize the accuracy of the pulse detection devices. For instance, issues related to noise disruption and environmental factors continue to hinder the detection accuracy of these devices (Ardila et al., 2019). Fortunately, machine learning presents an extensive potential to overcome these obstacles. The forthcoming pulse detection devices of the next-generation, underpinned by TENG, can significantly elevate signal sensitivity and precision through the utilization of pliable bioelectronic materials, algorithm optimization, and the integration of advanced models. Further, redesigning the device’s structure and adopting state-of-the-art manufacturing technology are crucial steps for commercial uses, as they can help overcome existing integration limitations (Su et al., 2016). Implementing these improvements can foster accurate decision-making and efficient healthcare delivery. Nonetheless, relying solely on machine learning-powered systems is inadequate to provide precise treatment (Li et al., 2019). Collecting patients’ lifestyle data via intelligent interaction and survey systems is necessary for obtaining more accurate treatment (Yao et al., 2022). Consequently, machine learning can perpetually refine its suggestions by incorporating fresh data and iteratively updating its algorithms.

Several new mathematical tools and signal processing techniques can assist in processing pulse signals accurately, however, there is currently a shortage of disease sample databases that corresponds with pulse signals (Tran et al., 2018). The establishment of such a database stands as an urgent and pivotal endeavor. At present, the available case samples remain confined to a limited scale, lacking the requisite representativeness and generality (King, 2009). Collaborating with hospitals to create pulse signal and disease databases is an efficient way to address this challenge. Such databases are not only propitious for research but also have commercial value. In spite of certain strides made within the domain, a number of challenges persist. Firstly, acquiring precise pulse data is difficult due to the complexity of the human cardiovascular system, and people’s situations vary. Secondly, the tester’s position of the brachial and radial arteries varies, posing a challenge in positioning the pulse wave sensor which affects measurement accuracy (Milner et al., 1991). In addition, existing algorithms primarily cater to normal populations and rely on data from typical resting and exercising states (Guo et al., 2023). Thus, test results may not be ideal for hypertensive patients or people under different physiological conditions such as alcohol consumption.

To overcome these challenges, an in-depth investigation of sensor acquisition is imperative. Long-term tracking of a large number of hypertensive patients and healthy individuals in diverse states must also be conducted (Venugopal et al., 2021). Enhancements in instruments and algorithmic adjustments can be perpetually refined through comprehensive analysis of survey statistical outcomes, culminating in heightened performance outputs (Rasel and Park, 2017). Despite the notable advancements in medical detection and clinical diagnosis using pulse detection devices based on TENG technology, several challenges persist (Wainwright et al., 2003). A combination of strategies such as machine learning, device structure modification, manufacturing technology alterations (Li et al., 2021b), and large-scale database development that correspond to pulse signals and diseases can harness the enormous potential of these technologies for efficient healthcare delivery and high-precision clinical decision-making (Xu et al., 2018b). Nevertheless, apprehensions surrounding pulse signal acquisition and algorithm applicability pose challenges in attaining heightened detection accuracy (Zou et al., 2020). Continuous improvement and thorough research can further promote the development of TENG-based pulse detection devices, and subsequently improve healthcare delivery and support for people’s wellbeing (Zheng et al., 2022; Ge et al., 2023).

## References

[B1] Ardila D. Kiraly A. P. Bharadwaj S. Choi B. Reicher J. J. Peng L. (2019). End-to-end lung cancer screening with three-dimensional deep learning on low-dose chest computed tomography. Nat. Med. 25 (6), 954–961. 10.1038/s41591-019-0447-x 31110349

[B2] Chen G. Li Y. Bick M. Chen J. (2020). Smart textiles for electricity generation. Chem. Rev. 120 (8), 3668–3720. 10.1021/acs.chemrev.9b00821 32202762

[B3] Cheng T. Gao Q. Wang Z. L. (2019). The current development and future outlook of triboelectric nanogenerators: a survey of literature. Adv. Mater. Technol. 4 (3), 1800588. 10.1002/admt.201800588

[B4] Fan F. R. Tian Z. Q. Wang Z. L. (2012). Flexible triboelectric generator. Nano energy 1 (2), 328–334. 10.1016/j.nanoen.2012.01.004

[B5] Ge X. Gao Z. Zhang L. Ji H. Yi J. Jiang P. (2023). Flexible microfluidic triboelectric sensor for gesture recognition and information encoding. Nano Energy 113, 108541. 10.1016/j.nanoen.2023.108541

[B6] Guo X. He J. Zheng Y. Wu J. Pan C. Zi Y. (2023). High-performance triboelectric nanogenerator based on theoretical analysis and ferroelectric nanocomposites and its high-voltage applications. Nano Res. Energy 2 (3), e9120074. 10.26599/nre.2023.9120074

[B7] He X. Zou H. Geng Z. Wang X. Ding W. Hu F. (2018). A hierarchically nanostructured cellulose fiber-based triboelectric nanogenerator for self-powered healthcare products. Adv. Funct. Mater. 28 (45), 1805540. 10.1002/adfm.201805540

[B8] Jiang D. Lian M. Xu M. Sun Q. Xu B. B. Thabet H. K. (2023). Advances in triboelectric nanogenerator technology—applications in self-powered sensors, Internet of things, biomedicine, and blue energy. Adv. Compos. Hybrid Mater. 6 (2), 57. 10.1007/s42114-023-00632-5

[B9] Khandelwal G. Minocha T. Yadav S. K. Chandrasekhar A. Maria Joseph Raj N. P. Gupta S. C. (2019). All edible materials derived biocompatible and biodegradable triboelectric nanogenerator. Nano Energy 65, 104016. 10.1016/j.nanoen.2019.104016

[B10] King D. E. (2009). Dlib-ml: A machine learning toolkit. J. Mach. Learn. Res. 10, 1755–1758.

[B11] Lai Y. C. Deng J. Zhang S. L. Niu S. Guo H. Wang Z. L. (2017). Single-thread-based wearable and highly stretchable triboelectric nanogenerators and their applications in cloth-based self-powered human-interactive and biomedical sensing. Adv. Funct. Mater. 27 (1), 1604462. 10.1002/adfm.201604462

[B12] Leng Z. Zhu P. Wang X. Wang Y. Li P. Huang W. (2023). Sebum-membrane-Inspired protein-based bioprotonic hydrogel for artificial skin and human-machine merging interface. Adv. Funct. Mater. 33 (13), 2211056. 10.1002/adfm.202211056

[B13] Li G. Liu Y. Chen Y. Li M. Song J. Li K. (2023a). Polyvinyl alcohol/polyacrylamide double-network hydrogel-based semi-dry electrodes for robust electroencephalography recording at hairy scalp for noninvasive brain–computer interfaces. J. Neural Eng. 20 (2), 026017. 10.1088/1741-2552/acc098 36863014

[B14] Li G. Liu C. Zou H. Che L. Sun P. Yan J. (2023b). Integrated wearable smart sensor system for real-time multi-parameter respiration health monitoring. Cell Rep. Phys. Sci. 4 (1), 101191. 10.1016/j.xcrp.2022.101191

[B15] Li G. Wang S. Li M. Duan Y. Y. (2021a). Towards real-life EEG applications: novel superporous hydrogel-based semi-dry EEG electrodes enabling automatically ‘charge–discharge’electrolyte. J. Neural Eng. 18 (4), 046016. 10.1088/1741-2552/abeeab 33721854

[B16] Li G. Dai K. Zhang W. Wang X. You Z. Zhang H. (2021b). Triboelectric nanogenerator-based wearable electronic devices and systems: toward informatization and intelligence. Digit. Signal Process. 113, 103038. 10.1016/j.dsp.2021.103038

[B17] Li X. Lau T. H. Guan D. Zi Y. (2019). A universal method for quantitative analysis of triboelectric nanogenerators. J. Mater. Chem. A 7 (33), 19485–19494. 10.1039/c9ta06525c

[B18] Lin Z. Zhang B. Zou H. Wu Z. Guo H. Zhang Y. (2020). Rationally designed rotation triboelectric nanogenerators with much extended lifetime and durability. Nano Energy 68, 104378. 10.1016/j.nanoen.2019.104378

[B19] Liu D. Zhu P. Zhang F. Li P. Huang W. Li C. (2023). Intrinsically stretchable polymer semiconductor based electronic skin for multiple perceptions of force, temperature, and visible light. Nano Res. 16 (1), 1196–1204. 10.1007/s12274-022-4622-x

[B20] Liu W. Liu W. Wang Z. He W. Tang Q. Xi Y. (2020a). Quantifying contact status and the air-breakdown model of charge-excitation triboelectric nanogenerators to maximize charge density. Nat. Commun. 11 (1), 1599. 10.1038/s41467-020-15368-9 32221300 PMC7101333

[B21] Liu W. Wang Z. Wang G. Zeng Q. He W. Liu L. (2020b). Switched-capacitor-convertors based on fractal design for output power management of triboelectric nanogenerator. Nat. Commun. 11 (1), 1883. 10.1038/s41467-020-15373-y 32312950 PMC7171113

[B22] Liu W. Wang Z. Wang G. Zeng Q. He W. Liu L. (2020c). Switched-capacitor-convertors based on fractal design for output power management of triboelectric nanogenerator. Nat. Commun. 11 (1), 1883. 10.1038/s41467-020-15373-y 32312950 PMC7171113

[B23] Liu Z. Zhao Z. Zeng X. Fu X. Hu Y. (2019). Expandable microsphere-based triboelectric nanogenerators as ultrasensitive pressure sensors for respiratory and pulse monitoring. Nano Energy 59, 295–301. 10.1016/j.nanoen.2019.02.057

[B24] Lv J. Shao X. Xing J. (2017). “A deep regression architecture with two-stage re-initialization for high performance facial landmark detection,” in Proceedings of the IEEE conference on computer vision and pattern recognition, Honolulu, HI, USA, 21-26 July 2017 (IEEE), 3317–3326.

[B25] Meng B. Tang W. Zhang X. Han M. Liu W. Zhang H. (2013). Self-powered flexible printed circuit board with integrated triboelectric generator. Nano Energy 2 (6), 1101–1106. 10.1016/j.nanoen.2013.08.006

[B26] Meng K. Chen J. Li X. Wu Y. Fan W. Zhou Z. (2019). Flexible weaving constructed self-powered pressure sensor enabling continuous diagnosis of cardiovascular disease and measurement of cuffless blood pressure. Adv. Funct. Mater. 29 (5), 1806388. 10.1002/adfm.201806388

[B27] Milner R. Tofte M. Harper R. (1991). Commentary on standard ML. Cambridge, Massachusetts: MIT press.

[B28] Ouyang H. Tian J. Sun G. Zou Y. Liu Z. Li H. (2017). Self-powered pulse sensor for antidiastole of cardiovascular disease. Adv. Mater. 29 (40), 1703456. 10.1002/adma.201703456 28863247

[B29] Rasel M. S. U. Park J. Y. (2017). A sandpaper assisted micro-structured polydimethylsiloxane fabrication for human skin based triboelectric energy harvesting application. Appl. Energy 206, 150–158. 10.1016/j.apenergy.2017.07.109

[B30] Riera A. Soria-Frisch A. Albajes-Eizagirre A. Cipresso P. Grau C. Dunne S. (2012). Electro-physiological data fusion for stress detection. Annu. Rev. Cybertherapy Telemedicine 181, 228–232. 22954861

[B31] Seo D. Neely R. M. Shen K. Singhal U. Alon E. Rabaey J. M. (2016). Wireless recording in the peripheral nervous system with ultrasonic neural dust. Neuron 91 (3), 529–539. 10.1016/j.neuron.2016.06.034 27497221

[B32] Su Y. Xie G. Chen J. Du H. Zhang H. Yuan Z. (2016). Reduced graphene oxide–polyethylene oxide hybrid films for toluene sensing at room temperature. RSC Adv. 6 (100), 97840–97847. 10.1039/c6ra21077e

[B33] Suguna G. C. Veerabhadrappa S. T. (2019). A review of wrist pulse analysis. Biomed. Res. 30 (4), 538–545. 10.35841/biomedicalresearch.30-19-174

[B34] Tran B. Q. Miller P. R. Taylor R. M. Boyd G. Mach P. M. Rosenzweig C. N. (2018). Proteomic characterization of dermal interstitial fluid extracted using a novel microneedle-assisted technique. J. proteome Res. 17 (1), 479–485. 10.1021/acs.jproteome.7b00642 29172549

[B35] Venugopal K. Panchatcharam P. Chandrasekhar A. Shanmugasundaram V. (2021). Comprehensive review on triboelectric nanogenerator based wrist pulse measurement: sensor fabrication and diagnosis of arterial pressure. ACS sensors 6 (5), 1681–1694. 10.1021/acssensors.0c02324 33969980

[B36] Wainwright M. J. Jaakkola T. S. Willsky A. S. (2003). “Tree-reweighted belief propagation algorithms and approximate ML estimation by pseudo-moment matching,” in International Workshop on Artificial Intelligence and Statistics, 08 January 2003 (PMLR), 308–315.

[B37] Wang S. Xie Y. Niu S. Lin L. Liu C. Zhou Y. S. (2014). Maximum surface charge density for triboelectric nanogenerators achieved by ionized-air injection: methodology and theoretical understanding. Adv. Mater. 26 (39), 6720–6728. 10.1002/adma.201402491 25146891

[B38] Wang Z. L. Chen J. Lin L. (2015). Progress in triboelectric nanogenerators as a new energy technology and self-powered sensors. Energy and Environ. Sci. 8 (8), 2250–2282. 10.1039/c5ee01532d

[B39] Wang Z. L. (2017). On maxwell's displacement current for energy and sensors: the origin of nanogenerators. Mater. Today 20 (2), 74–82. 10.1016/j.mattod.2016.12.001

[B40] Wang Z. L. (2020). On the first principle theory of nanogenerators from Maxwell's equations. Nano Energy 68, 104272. 10.1016/j.nanoen.2019.104272

[B41] Wu Z. Zhang B. Zou H. Lin Z. Liu G. Wang Z. L. (2019). Multifunctional sensor based on translational-rotary triboelectric nanogenerator. Adv. Energy Mater. 9 (33), 1901124. 10.1002/aenm.201901124

[B42] Xiong J. Lee P. S. (2019). Progress on wearable triboelectric nanogenerators in shapes of fiber, yarn, and textile. Sci. Technol. Adv. Mater. 20 (1), 837–857. 10.1080/14686996.2019.1650396 31497178 PMC6720508

[B43] Xu C. Wang A. C. Zou H. Zhang B. Zhang C. Zi Y. (2018a). Raising the working temperature of a triboelectric nanogenerator by quenching down electron thermionic emission in contact-electrification. Adv. Mater. 30 (38), 1803968. 10.1002/adma.201803968 30091484

[B44] Xu C. Zi Y. Wang A. C. Zou H. Dai Y. He X. (2018b). On the electron-transfer mechanism in the contact-electrification effect. Adv. Mater. 30 (15), 1706790. 10.1002/adma.201706790 29508454

[B45] Xu Q. Fang Y. Jing Q. Hu N. Lin K. Pan Y. (2021). A portable triboelectric spirometer for wireless pulmonary function monitoring. Biosens. Bioelectron. 187, 113329. 10.1016/j.bios.2021.113329 34020223 PMC8118703

[B46] Xu Z. Bao K. Di K. Chen H. Tan J. Xie X. (2022). High-performance dielectric elastomer nanogenerator for efficient energy harvesting and sensing via alternative current method. Adv. Sci. 9 (18), 2201098. 10.1002/advs.202201098 PMC9218771 35396790

[B47] Yang J. Chen J. Su Y. Jing Q. Li Z. Yi F. (2015). Eardrum-inspired active sensors for self-powered cardiovascular system characterization and throat-attached anti-interference voice recognition. Adv. Mater. 27 (8), 1316–1326. 10.1002/adma.201404794 25640534

[B48] Yao S. Zhao X. Wang X. Huang T. Ding Y. Zhang J. (2022). Bioinspired electron polarization of nanozymes with a human self-generated electric field for cancer catalytic therapy. Adv. Mater. 34 (15), 2109568. 10.1002/adma.202109568 35151235

[B49] Yu A. Zhu Y. Wang W. Zhai J. (2019). Progress in triboelectric materials: toward high performance and widespread applications. Adv. Funct. Mater. 29 (41), 1900098. 10.1002/adfm.201900098

[B50] Yuan M. Li C. Liu H. Xu Q. Xie Y. (2021). A 3D-printed acoustic triboelectric nanogenerator for quarter-wavelength acoustic energy harvesting and self-powered edge sensing. Nano Energy 85, 105962. 10.1016/j.nanoen.2021.105962

[B51] Zeng Z. Huang Z. Leng K. Han W. Niu H. Yu Y. (2020). Nonintrusive monitoring of mental fatigue status using epidermal electronic systems and machine-learning algorithms. ACS sensors 5 (5), 1305–1313. 10.1021/acssensors.9b02451 31939287

[B52] Zhang H. Yang Y. Su Y. Chen J. Hu C. Wu Z. (2013). Triboelectric nanogenerator as self-powered active sensors for detecting liquid/gaseous water/ethanol. Nano Energy 2 (5), 693–701. 10.1016/j.nanoen.2013.08.004

[B53] Zhang J. Xu Q. Li H. Zhang S. Hong A. Jiang Y. (2022). Self-powered electrodeposition system for sub-10-nm silver nanoparticles with high-efficiency antibacterial activity. J. Phys. Chem. Lett. 13 (29), 6721–6730. 10.1021/acs.jpclett.2c01737 35849530

[B54] Zhang N. Tao C. Fan X. Chen J. (2017). Progress in triboelectric nanogenerators as self-powered smart sensors. J. Mater. Res. 32 (9), 1628–1646. 10.1557/jmr.2017.162

[B55] Zhang Z. Luo P. Loy C. C. (2014). “Facial landmark detection by deep multi-task learning,” in Proceedings, Part VI 13 Computer Vision–ECCV 2014: 13th European Conference, Zurich, Switzerland, September 6-12, 2014 (Berlin, Germany: Springer International Publishing), 94–108.

[B56] Zheng Y. Liu T. Wu J. Xu T. Wang X. Han X. (2022). Energy conversion analysis of multilayered triboelectric nanogenerators for synergistic rain and solar energy harvesting. Adv. Mater. 34 (28), 2202238. 10.1002/adma.202202238 35538660

[B57] Zhou H. Huang W. Xiao Z. Zhang S. Li W. Hu J. (2022). Deep-learning-assisted noncontact gesture-recognition system for touchless human-machine interfaces. Adv. Funct. Mater. 32 (49), 2208271. 10.1002/adfm.202208271

[B58] Zhou T. Zhang C. Han C. B. Fan F. R. Tang W. Wang Z. L. (2014). Woven structured triboelectric nanogenerator for wearable devices. ACS Appl. Mater. interfaces 6 (16), 14695–14701. 10.1021/am504110u 25065506

[B59] Zou H. Guo L. Xue H. Zhang Y. Shen X. Liu X. (2020). Quantifying and understanding the triboelectric series of inorganic non-metallic materials. Nat. Commun. 11 (1), 2093. 10.1038/s41467-020-15926-1 32350259 PMC7190865

[B60] Zou H. Zhang Y. Guo L. Wang P. He X. Dai G. (2019). Quantifying the triboelectric series. Nat. Commun. 10 (1), 1427. 10.1038/s41467-019-09461-x 30926850 PMC6441076

